# The CD81 Partner EWI-2wint Inhibits Hepatitis C Virus Entry

**DOI:** 10.1371/journal.pone.0001866

**Published:** 2008-04-02

**Authors:** Vera Rocha-Perugini, Claire Montpellier, David Delgrange, Czeslaw Wychowski, François Helle, André Pillez, Hervé Drobecq, François Le Naour, Stéphanie Charrin, Shoshana Levy, Eric Rubinstein, Jean Dubuisson, Laurence Cocquerel

**Affiliations:** 1 Institut de Biologie de Lille (UMR8161), CNRS, Universités de Lille I et Lille II, Institut Pasteur de Lille, Lille, France; 2 Division of Oncology, Department of Medicine, Stanford University Medical Center, Stanford, California, United States of America; 3 INSERM-U602, Institut André-Lwoff, Université Paris XI, Hôpital Paul Brousse, Villejuif, France; The Rockefeller University, United States of America

## Abstract

Two to three percent of the world's population is chronically infected with hepatitis C virus (HCV) and thus at risk of developing liver cancer. Although precise mechanisms regulating HCV entry into hepatic cells are still unknown, several cell surface proteins have been identified as entry factors for this virus. Among these molecules, the tetraspanin CD81 is essential for HCV entry. Here, we have identified a partner of CD81, EWI-2wint, which is expressed in several cell lines but not in hepatocytes. Ectopic expression of EWI-2wint in a hepatoma cell line susceptible to HCV infection blocked viral entry by inhibiting the interaction between the HCV envelope glycoproteins and CD81. This finding suggests that, in addition to the presence of specific entry factors in the hepatocytes, the lack of a specific inhibitor can contribute to the hepatotropism of HCV. This is the first example of a pathogen gaining entry into host cells that lack a specific inhibitory factor.

## Introduction

Hepatitis C virus (HCV) infection is a global public health problem affecting over 130 million individuals worldwide; its symptoms including chronic hepatitis, liver cirrhosis, and hepatocellular carcinoma [1]. Unfortunately, no vaccine is currently available to prevent new infections and the current treatments are not fully efficient [2]. Clearly, new therapeutic strategies are urgently required.

Over the past decade, due to the lack of a cell culture system supporting production of infectious virus particles, several surrogate models have been developed to facilitate analysis of the HCV life cycle. Among these models, pseudoparticles (HCVpp), consisting of native HCV envelope glycoproteins assembled onto retroviral core particles [3], [4] have been useful in investigating the HCV entry process. More recently, however, production of infectious HCV particles in cell culture (HCVcc) has finally become possible [5], [6], [7]. This powerful system is based on the transfection of the human hepatoma cell line Huh-7 with the cloned JFH1 genome that replicates and produces infectious particles.

HCV encodes two envelope glycoproteins, E1 and E2, that interact to form a noncovalent E1E2 heterodimer [8] which is present at the surface of HCV particles [6], and is therefore the obvious candidate ligand for cellular receptor(s). Although the early steps of viral entry have yet to be elucidated, several cell-surface expressed molecules have been proposed as entry factors for HCV (reviewed in [9]). Among these molecules, the tetraspanin CD81 and the scavenger receptor class B type I (SR-BI) have been shown to play major roles in HCV entry. However, co-expression of these two molecules in non-hepatic cell lines does not lead to HCV entry [10], suggesting that additional molecule(s) are involved in control of HCV entry. Recently, the tight-junction components Claudins (CLDN-1, CLDN-6, CLDN-9) have been identified as additional key factors for HCV infection [11], [12]. Interestingly, CLDN-1 is the first entry factor shown to confer susceptibility to HCV when ectopically expressed in non-hepatic cells. Nevertheless, although CLDN-1 subcellular distribution seems to modulate HCV permissivity [13], some human cell lines expressing CD81, SR-BI and CLDN-1 remain resistant to HCV entry suggesting that one or more human-specific HCV entry factor(s) remain to be discovered [11].

CD81 belongs to the tetraspanin family. Members of this family organize and regroup their associated transmembrane proteins and are involved in various functions such as cell morphology, motility, fusion and signalling [14], [15]. A major characteristic of tetraspanins is their ability to interact with each other and with other transmembrane proteins, thus building membrane multi-molecular complexes, collectively referred to as the tetraspanin web [16], [17]. Within this network of interactions, tetraspanins form primary complexes with a limited number of proteins termed tetraspanin partners. These primary interactions are direct, highly specific and occur at high stoichiometry. Two major partners have been identified for CD81, EWI-F (also called CD9P-1, FPRP or CD315) and EWI-2 (also called PGRL, IgSF8 or CD316) [18], [19], [20], [21], [22], which may provide a link between the tetraspanin web and the actin cytoskeleton by interacting with Ezrin, an Ezrin-Radixin-Moesin (ERM) protein [23]. Although its function is still unclear, EWI-2 seems to participate in the regulation of cellular functions such as aggregation, spreading, motility and migration [24], [25], [26].

In this work, we identified a cleavage product of EWI-2, which associates with CD81 and inhibits its interaction with the HCV envelope glycoproteins. Most importantly, this molecule, that we called EWI-2wint (EWI-2 without its N-terminus), has an inhibitory effect on HCV entry, highlighting a potential new mechanism for the regulation of cellular invasion by this pathogen.

## Results

### A CD81 partner blocks the interaction between CD81 and HCV envelope glycoproteins

**Figure 1:**
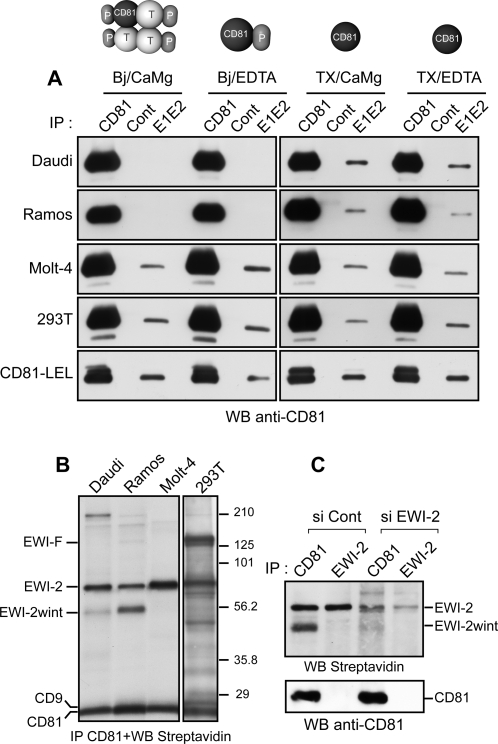
The interaction between HCV glycoproteins and CD81 is blocked when CD81 interacts with partner protein(s). A, HCV E1E2 heterodimers immobilized onto anti-E2 coated beads interacted with CD81 in all cells lysed in Triton X-100 (TX). However, the interaction of HCV-E1E2 with CD81 was blocked in Daudi and Ramos cells lysed in Brij (Bj). Maintenance of CD81 with other tetraspanins (T) and partners (P) is disrupted by the indicated lysis conditions, as diagrammed. Anti-CD81 and irrelevant (Cont) mAbs were used in immunoprecipitations as controls. Precipitation of CD81 was revealed by western blotting with the anti-CD81 5A6 mAb. CD81-LEL corresponds to the large extracellular loop of CD81 fused to the glutathione-S transferase. B, After cell surface biotinylation, the indicated cell lines were lysed with Bj/EDTA, immunoprecipitated with 5A6 mAb and the proteins revealed by Western blotting with HRP-conjugated streptavidin. The values on the right are molecular sizes in kilodaltons. C, EWI-2wint production is directly connected to EWI-2 expression. Daudi cells interfered with negative siRNA or EWI-2 siRNA were biotinylated, lysed in Bj/EDTA, immunoprecipitated with the anti-CD81 5A6 mAb or an anti-EWI-2 mAb (8A12) and blotted sequentially with HRP-conjugated streptavidin and 5A6 mAb.

Tetraspanin microdomains are typically disrupted by Triton X-100 (TX), but are retained in less hydrophobic detergents such as Brij97 (Bj). In addition, the replacement of divalent cations (CaMg) by EDTA in the Brij97 lysis buffer causes a disruption of tetraspanin/tetraspanin interactions but conserves the tetraspanin/partner interactions [18] (Figure 1A). These biochemical properties allowed us to investigate whether the interaction between CD81 and E1E2 heterodimers is similar when CD81 is embedded or not in a tetraspanin web or in a primary complex. For this purpose, we analysed the interaction between E1E2 heterodimers and CD81 in several cell lines lysed in different detergent conditions (Figure 1A and Table 1). The E1E2 complexes were immobilized on agarose beads bound to a conformation-sensitive monoclonal antibody (mAb H53). Analysis of these agarose-bound complexes under non-reducing conditions [27] showed proper folding of the heterodimers in the different detergent conditions (data not shown). As positive and negative controls, we used anti-CD81 and irrelevant mouse (Cont) mAbs, respectively. A recombinant soluble form of the CD81 large extracellular loop (CD81-LEL) [28] was also used as a control. CD81 from Molt-4 and 293T cells interacted with E1E2 heterodimers under all detergent conditions, as did the CD81-LEL (Figure 1A). In contrast, CD81 from Daudi and Ramos cells did not interact with E1E2 heterodimers when tetraspanin webs or primary complexes were conserved (Figure 1A, Bj/CaMg and Bj/EDTA, respectively). Daudi cells also did not react with E1E2 glycoproteins derived from HCVpp (data not shown). Together, our results suggest that a CD81 partner, present on Daudi and Ramos cells, blocks the interaction between CD81 and HCV glycoproteins. Testing of other cell lines indicated that such a CD81 partner is probably also present in other cell lines such as A431, a squamous carcinoma cell line (Table 1).

**Table 1:** *In vitro* interaction of HCV E1E2 heterodimers with CD81 and expression of cell markers.

Cell lines	E1E2/CD81 interaction^1^	SR-BI^2^	Claudin-1^2^	CD81^3^	EWI-2^3^	EWI-2wint^4^
Hematopoietic cell lines						
B cells						
Daudi	-	++	-	+	+	+
Ramos	-	++	-	+	+	+
OCI-LY8	+/−	++	-	+	+	+/−
T cells						
Jurkat	+/−	+/−	-	+	+	+/−
Molt-4	+	+/−	-	+	+	-
Myeloid cells			-			
U937	-	+/−	-	-	-	-
U937-CD81	+	+/−	-	+	-	-
PBMC	+/−	ND	ND	+	+	+/−
Non Hematopoietic cell lines						
Fetal kidney, 293T	+	+	-	+	+	-
Squamous carcinoma, A431	-	+	+	+	+	+
Hepatocyte carcinoma						
Huh-7	+	++	+	+	+	-
HepG2-CD81	ND	ND	ND	+	+	-
PLC/PRF-5	ND	ND	ND	+	+	-
Primary hepatocytes	+	+	+	+	+	-

1 The ratio [CD81 band intensity in E1E2 IP in Bj/EDTA buffer]/[CD81 band intensity in E1E2 IP in TX/EDTA buffer] was used to evaluate the E1E2/CD81 interaction: -, ≤0.1; +/−, >0.1–0.7<; +,≥0.7

2 Expression of SR-BI and Claudin-1 detected by WB with Cla-1 mAb and JAY.8 polyclonal Ab, respectively. Amount of each protein was compared to Huh-7 cells. The ratio [protein band intensity of the cell line]/[protein band intensity of Huh-7 cell line] was used: -, <0.1; +/−, ≥0.1–0.4<; +, ≥0.4–1<; ++, ≥1. Actin proteins were used for normalization.

3 Cell surface expression of CD81 and EWI-2 proteins detected by flow cytometry with 5A6 and 8A12 mAbs, respectively. Analyses gave mean fluorescence intensities (MFI) in the range of 3–6 for negative controls and intensities over 10 for specific stainings.

4 Expression detected by co-immunoprecipitation with CD81 as described in Figure 1B. The ratio [EWI-2wint band intensity]/[CD81 band intensity] was used to evaluate the expression of EWI-2wint: -, ≤0.01; +/−, >0.01–0.1≤; +, >0.1

To identify the CD81 partner blocking the interaction between CD81 and E1E2, we performed co-immunoprecipitation experiments using Bj/EDTA lysates from surface biotinylated cells. Under these conditions, only partners directly associated in primary complexes co-precipitate with CD81. In all cell lines, a 70-kDa surface protein, corresponding to EWI-2 [18], [21] was co-precipitated with CD81 (Figure 1B). EWI-F, the second major CD81/CD9 partner [19], [22] was also co-precipitated with CD81 and CD9 in 293T cells. To confirm the specificity of EWI-2 and EWI-F, these proteins were immunoprecipitated with specific mAbs 8A12 and 1F11, respectively (data not shown). Additional bands were also observed in these cells, but the identity of the corresponding proteins is unknown. Interestingly, an additional ∼55 kDa surface protein co-precipitated with CD81 in Daudi and Ramos cells but not in Molt-4 and 293T cells. This protein, which we have named EWI-2wint, has been previously proposed as a cleavage product of EWI-2 that remains associated with CD81 and CD9 [18], [21]. Furthermore, transfection of EWI-2 siRNA into Daudi cells reduced both the expression of EWI-2 and of EWI-2wint (Figure 1C) demonstrating that EWI-2wint production is directly connected to EWI-2 expression. We performed additional co-immunoprecipitation experiments using different surface biotinylated cell lysates (Table 1). Co-immunoprecipitation of EWI-2wint with CD81 correlated with the inhibition of CD81-E1E2 interaction in mild detergents. Conversely, cells for which CD81 bound to E1E2 did not express this protein. It is worth noting that primary hepatocytes and hepatoma cells do not have detectable levels of EWI-2wint. We also analysed SR-BI and CLDN-1 expression in the same cell lines, and the lack of interaction between CD81 and E1E2 glycoproteins cannot be correlated with the absence of these molecules (Table 1, see squamous carcinoma, A431). Altogether, our results suggest that EWI-2wint probably corresponds to the CD81 partner blocking the interaction between CD81 and HCV glycoproteins.

### EWI-2wint corresponds to EWI-2 with its amino-terminus deleted

**Figure 2:**
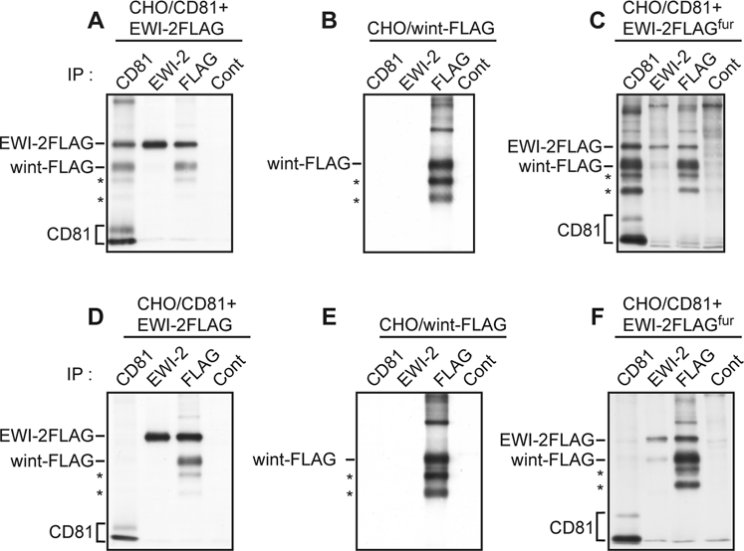
EWI-2wint is a cleavage product of EWI-2. CHO cells were (co)-transfected with pcDNA3.1/CD81 and pcDNA3.1/EWI-2FLAG (A and D), with pcDNA3.1/wint-FLAG (B and E), or with pcDNA3.1/CD81 and pcDNA3.1/EWI-2FLAG^fur^ (C and F). EWI-2FLAG^fur^ corresponds to EWI-2FLAG in which a furin cleavage site has been engineered. After cell surface biotinylation, cells were lysed with Bj/EDTA (A–C) or TX/EDTA (D–F) and proteins were immunoprecipitated with the indicated mAbs. Proteins were revealed by Western blotting with HRP-conjugated streptavidin. Asterisks indicate additional cleavage products of EWI-2.

To confirm that EWI-2wint is a cleavage product of EWI-2, we constructed an EWI-2 cDNA with a C-terminal FLAG epitope tag (EWI-2FLAG), as described previously [21]. CHO cells stably co-expressing human CD81 and EWI-2FLAG (CHO/CD81+EWI-2FLAG) were surface biotinylated, lysed in Bj/EDTA (Figure 2A) or TX/EDTA (Figure 2D) and analysed in immunoprecipitation experiments. Upon EWI-2FLAG coexpression with CD81, EWI-2wint was detected following anti-FLAG immunoprecipitation or co-immunoprecipitation with CD81 (wint-FLAG ; Figure 2A). These results and Western blotting analyses (data not shown) showed that EWI-2wint still contains the C-terminal FLAG epitope, making EWI-2wint an authentic cleavage product of EWI-2. It is interesting to note that EWI-2wint was not recognized by our anti-EWI-2 mAb and that only a small fraction of CD81 co-immunoprecipitated with EWI-2 products, as previously observed [18]. Two smaller proteins of ∼50, and 45 kDa, which remained associated with CD81 and probably correspond to additional cleavage products of EWI-2 were also detected (asterisks in Figure 2).

**Figure 3:**
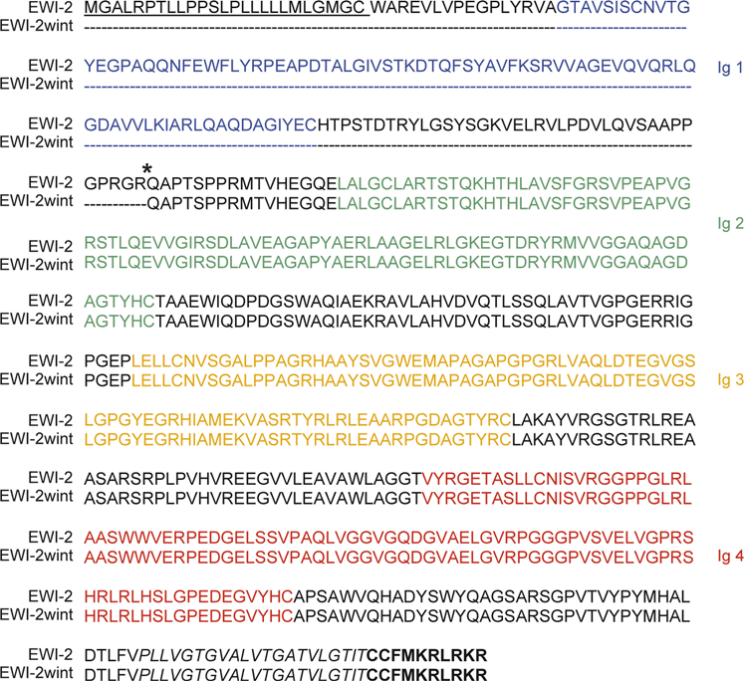
Alignment of EWI-2 sequence and the deduced EWI-2wint sequence. Asterisk indicates the position at which an Arg (R) residue was introduced to make a furin cleavage site. Underlined amino acids correspond to the signal peptide of EWI-2. The four Ig domains are colored. Amino acid residues corresponding to the putative transmembrane domain are in italics and those corresponding to the cytoplasmic tail are bolded.

EWI-2 belongs to a novel family of immunoglobulin (Ig) proteins, which also includes EWI-F/CD9P-1/PGRL, EWI-101/CD101/V7 and EWI-3/IgSF3 [21]. The members of this family share a conserved EWI motif and contain an ectodomain composed of V-type Ig domains, a transmembrane domain and a short highly charged cytoplasmic tail [21]. EWI-2 contains 4 Ig domains (Figure 3).

To identify the sequence of EWI-2wint, EWI-2FLAG and wint-FLAG proteins were purified by immunoaffinity from a Triton X-100 lysate of CHO/CD81+EWI-2FLAG and fractionated by SDS-PAGE. The bands corresponding to EWI-2FLAG and wint-FLAG were excised after colloidal blue staining and digested with trypsin or V8 endoproteinase Glu C and the resulting peptides analysed by mass spectrometry (Table 2). Interestingly, only peptides matching with the N-terminal membrane distal Ig domain (Ig 1 domain) of EWI-2 were not present in the wint-FLAG band, suggesting that this domain may be absent in the EWI-2wint protein. Indeed, sequencing of purified proteins by Edman degradation revealed that the N-terminal sequence of EWI-2wint corresponds to the QAPTS amino acids (Table 2) residues 166 to 170, which are located within the spacer separating the Ig1 and Ig2 domains of EWI-2 (Figure 3). Altogether, these results indicate that EWI-2wint corresponds to EWI-2 without its Ig 1 domain. Following these results, we chose to name this protein EWI-2wint for EWI-2 without its N-terminus.

**Table 2:** Mass spectrometric analyses and N-terminal sequencing of EWI-2 and EWI-2wint proteins

SEQUENCE	POSITION	MASS	ENZ	EWI-2	EWI-2wint
EVLVPEGPLYR	29–39	1271	Tryp	+	-
DTQFSYAVFK	83–92	1205	Tryp	+	-
VVAGEVQVQR	95–104	1084	Tryp	+	-
LQAQDAGIYECHTPSTDTR	117–135	2163	Tryp	+	-
VLPDVLQVSAAPPGPR	148–163	1615	Tryp	+	-
MTVHEGQELALGCLAR	174–189	1784	Tryp	+	+
MTVHEGQELALGCLAR	174–189	1800	Tryp	+	+
HTHLAVSFGR	195–204	1124	Tryp	+	+
SVPEAPVGR	205–213	911	Tryp	+	+
STLQEVVGIR	214–223	1101	Tryp	+	+
SDLAVEAGAPYAER	224–237	1448	Tryp	+	+
LAAGELR	238–244	729	Tryp	+	+
MVVGGAQAGDAGTYHCTAAEWIQDPDGSWAQIAEK	255–289	3690	Tryp	+	+
AVLAHVDVQTLSSQLAVTVGPGER	291–314	2447	Tryp	+	+
HAAYSVGWEMAPAGAPGPGR	338–357	1981	Tryp	+	+
HAAYSVGWEMAPAGAPGPGR	338–357	1997	Tryp	+	+
LVAQLDTEGVGSLGPGYEGR	358–377	2018	Tryp	+	+
LEAARPGDAGTYR	393–405	1376	Tryp	+	+
SRPLPVHVR	427–435	1060	Tryp	+	+
GGPPGLR	468–474	653	Tryp	+	+
LHSLGPEDEGVYHCAPSAWVQHADYSWYQAGSAR	531–564	3844	Tryp	+	+
AVVLKIARLQAQD	109–121	1424	Glu-C	+	-
CHTPSTDTRYLGSYSGKVE	127–145	2157	Glu-C	+	-
VVGIRSDLAVE	219–229	1157	Glu-C	+	+
GSWAQIAEKRAVLAHVD	281–297	1850	Glu-C	+	+
MAPAGAPGPGRLVAQLDTE	347–365	1866	Glu-C	+	+
AASARSRPLPVHVREE	422–437	1774	Glu-C	+	+
AVAWLAGGTVYRGE	443–456	1449	Glu-C	+	+
LGVRPGGGPVSVE	508–520	1223	Glu-C	+	+
LVGPRSHRLRLHSLGPEDE	521–539	2168	Glu-C	+	+
**N-term**				**REVLV**	**QAPTS**

Tryp: trypsine; Glu-C: V8 endoproteinase Glu C

### Expression of EWI-2wint in HCV target cells

**Figure 4:**
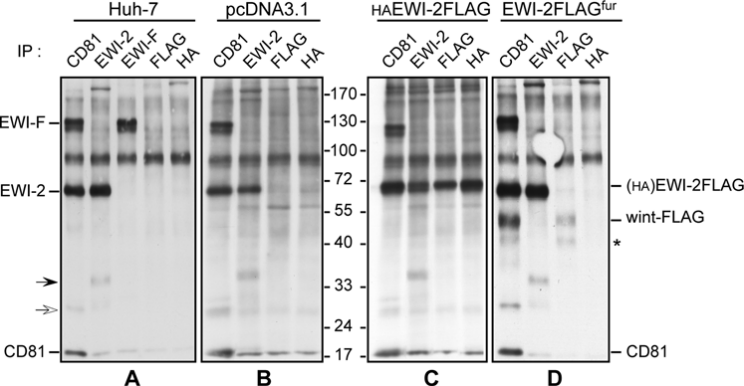
EWI-2wint expression in Huh-7 cells. After cell surface biotinylation, Huh-7 cells (A) stably expressing pcDNA3.1 (B), HAEWI-2FLAG (C) or EWI-2FLAG^fur^ (D) were lysed with Bj/EDTA and analyzed by immunoprecipitation with indicated mAbs. Proteins were revealed by Western blotting with HRP-conjugated streptavidin. The molecular weights of the prestained molecular ladder are indicated in KDa. The asterisk indicates an additional cleavage product of EWI-2. The band indicated by a light arrow likely corresponds to a dimer of CD81, and the band indicated by a full arrow to an unidentified EWI-2 associated protein.

To characterize EWI-2wint, we cloned it as a FLAG-tagged protein in an expression vector (pcDNA3.1/wint-FLAG). CHO cells transfected with pcDNA3.1/wint-FLAG produced EWI-2wint and the two additional cleavage products (asterisks), as shown in Figure 2B and 2E. To further test the role of EWI-2wint in HCV infection, we tried to obtain hepatoma cells (Huh-7) stably expressing EWI-2wint. However, despite several attempts, EWI-2wint expression in cellular clones could not be detected by cell surface biotinylation and immunoprecipitation or by western blotting experiments (data not shown), suggesting that this protein was poorly expressed in these cells. To circumvent this problem, we added a single Arg residue into the EWI-2FLAG sequence directly upstream to Q^166^ amino acid (asterisk, Figure 3), making an EWI-2FLAG protein with a RGRR cleavage motif for furin (pcDNA3.1/EWI-2FLAG^fur^), a trans-Golgi network associated endopeptidase. This strategy, when tested in CHO cells transfected with pcDNA3.1/EWI-2FLAG^fur^, yielded a high amount of the EWI-2wint protein, as shown in Figure 2C and 2F. Furthermore, EWI-2wint could be detected by direct immunoprecipitation or by co-immunoprecipitation with CD81 in Huh-7 cells stably expressing EWI-2FLAG^fur^ (wint-FLAG, Figure 4D). Almost no whole EWI-2FLAG protein reached the surface of Huh-7/EWI-2FLAG^fur^ cells, indicating that the cleavage of EWI-2FLAG^fur^ by furin was highly efficient in these cells. It should be noted that at least one additional cleavage product of EWI-2FLAG (asterisk, Figure 4D) was detected as in CHO cells. The association of one or two additional cleavage products with the production of EWI-2wint suggests that the first cleavage of EWI-2 allows the unmasking of one or two additional sites that are not accessible in the whole protein. In contrast, Huh-7 cells (Figure 4A), Huh-7 stably transfected with the empty vector (pcDNA3.1, Figure 4B) or with EWI-2FLAG, expressing a N-terminal HA epitope (HAEWI-2FLAG, Figure 4C) were devoid of EWI-2wint. In Huh-7 cells stably transfected with HAEWI-2FLAG, the tagged molecule was detected following co-immunoprecipitation with CD81 or by direct immunoprecipitation with anti-FLAG or anti-HA mAbs, as compared to control cells (Figure 4). The anti-CD81 and anti-FLAG antibodies precipitated different amounts of wint-FLAG in Huh-7/EWI-2FLAG^fur^ cells (Figure 4D). This might be due to differences in the affinity of the antibodies, as has been previously observed when EWI-2 and CD81 were coprecipitated [18]. Alternative possibilities are that i) homodimerization and heterodimerization of EWI-2 and CD81 modulate recognition by antibodies, as described [29], [30], ii) binding of the anti-FLAG mAb at the C-terminus of EWI-2wint induces a dissociation of the CD81/EWI-2wint complexes.

### EWI-2wint inhibits HCV infection

**Figure 5:**
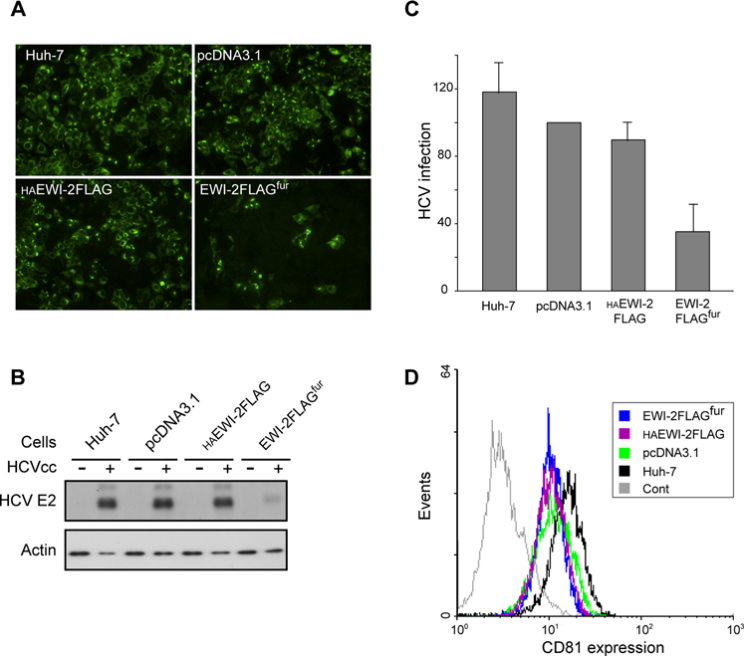
EWI-2wint inhibits HCVcc infection. Cell clones were infected with JFH1 HCVcc and infectivity was analyzed by indirect immunofluorescence with an anti-C mAb (A) or Western blotting with an anti-E2 mAb (B). The fields displayed in (A) contained similar numbers of cells. C, Infection levels were measured by quantification with NIH Image 1.62 of the intensities of HCV E2 bands. The results are presented as percentages of HCVcc infection relative to the infection of Huh-7/pcDNA3.1 cells. Infection levels of three independent experiments are reported as the mean with standard deviation bars. D, CD81 expression on the surfaces of cells expressing or not EWI-2wint. Cells were stained by using an anti-CD81 mAb (5A6) and secondary antibody conjugated with PE. Cont corresponds to Huh-7 cells that were stained only with the secondary antibody.

We then sought to determine whether EWI-2wint is able to modulate HCV infection of Huh-7 target cells. Originally we used cell culture produced HCV particles (HCVcc) [5], [6], [7] to infect Huh-7 cells expressing EWI-2FLAG^fur^, HAEWI-2FLAG, pcDNA3.1 or naïve Huh-7 cells. In the absence of a reporter gene in HCVcc, infection levels were evaluated by immunofluorescence (Figure 5A) and western blotting (Figure 5B), 40 h post-infection. Interestingly, we observed a reduction in HCVcc infection level in cells expressing EWI-2FLAG^fur^, whereas it remained unmodified in control cells (Figure 5C). It is worth noting that in HAEWI-2FLAG cells, the HCVcc infection level was similar to that of pcDNA3.1 cells, indicating that ectopic expression of HAEWI-2FLAG does not affect HCV infection. In addition, flow cytometry analyses ruled out any potential bias due to different levels of CD81 cell surface expression on clones (Figure 5D). Altogether, these results indicate that EWI-2wint produced in Huh-7/EWI-2FLAG^fur^ cells inhibits HCV infection.

### EWI-2wint blocks HCV entry into target cells

The life cycle of HCV can be divided into three major steps : entry of the virus into its target cells by receptor-mediated endocytosis, cytoplasmic and membrane-associated replication of the RNA genome, and assembly and release of the progeny virions.

**Figure 6:**
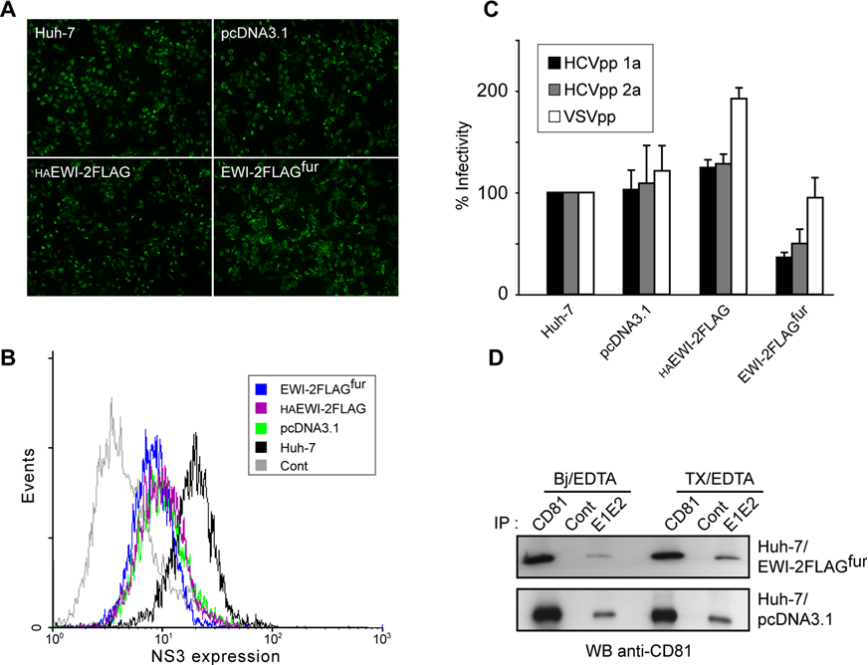
EWI-2wint inhibits the entry stage of HCV life cycle by reducing the interaction between E1E2 and CD81. A, EWI-2wint does not interfere with HCV replication. The indicated Huh-7 cell lines were transfected with the full-length JFH1 genome, and expression of the core antigen was evaluated at 40 h post-transfection. The displayed fields contained similar numbers of cells. B, NS3 expression in JFH-1 transfected cells. Cells were stained by using an anti-NS3 mAb (486-D39) and secondary antibody conjugated with PE. Cont corresponds to untransfected Huh-7 cells. C, Huh-7 cell lines were incubated for 3 h with virus pseudotyped with HCV envelope glycoprotein (HCVpp) or VSV G envelope protein (VSVpp). HCVpp were generated with envelope proteins from 1a (HCVpp 1a) or 2a (HCVpp 2a) genotype. The inoculum was then removed and the cells were further incubated. At 2 days post-inoculation, cells were lysed and processed to measure the luciferase activity. The luciferase activities were normalized for protein concentration in each cell lysate. The results are presented as relative percentages to HCVpp infectivity on Huh-7 cells. Results are reported as the mean±S.D. of three independent experiments. Pseudotyped particles produced in the absence of envelope proteins were used as controls. The mean fluorescence activity of such particles represented less than 2% of the activity measured for HCVpp. D, *In vitro* interaction of E1E2 heterodimers with CD81 from Huh-7/EWI-2FLAG^fur^ and Huh-7/pcDNA3.1. This assay was performed as described in Figure 1. The ratio [CD81 band intensity in E1E2 IP in Bj/EDTA buffer]/[CD81 band intensity in E1E2 IP in TX/EDTA buffer] was 0,27 and 0,84 in Huh-7/EWI-2FLAG^fur^ and Huh-7/pcDNA3.1, respectively.

To analyze the effect of EWI-2wint expression on HCV genome replication, we compared the efficiency of RNA replication and virus production between transfected cell lines expressing or lacking EWI-2wint. We transfected equal amounts of RNA corresponding to the full-length JFH1 genome [6] into the different cell lines (Figures 6A and 6B). Immunofluorescence analyses of transfected cells showed similar positivity for HCV anti-core antibody at 40 h post-transfection. Flow cytometry analyses using an anti-NS3 mAb showed that, although slightly stronger in Huh-7 cells, viral expression was similar in cellular clones that lacked or expressed EWI-2wint (Figure 6B), indicating that EWI-2wint does not affect HCV genome replication. Next, to assess the effect of EWI-2wint on the assembly and/or egress of particles, the culture medium of each transfected cell line was harvested and used to infect naïve Huh-7 cells. Through core-specific immunofluorescence staining, we found that Huh-7 cells were similarly infected by virus produced from all transfected cellular clones (data not shown), suggesting that EWI-2wint does not affect HCV assembly and release.

Our observations indicate that EWI-2wint likely affects an early step in the HCV life cycle. To test this hypothesis, we analyzed the effect of EWI-2wint on the infectivity of retroviral particles pseudotyped with HCV E1E2 proteins (Figure 6C). Use of HCVpp enables the analysis of the HCV entry step. We generated particles pseudotyped with HCV envelope glycoproteins from 1a and 2a genotypes (HCVpp 1a and HCVpp 2a, respectively) and control particles pseudotyped with the envelope glycoprotein G of vesicular stomatitis virus (VSVpp). As shown in Figure 6C, HCVpp infectivity was reduced in cells expressing EWI-2wint whereas it remained unmodified in control cells. Expression of EWI-2wint in target cells had no effect on VSVpp infectivity. It is interesting to note that HAEWI-2FLAG expression slightly increased HCVpp and VSVpp infectivity. Altogether, our results indicate that EWI-2wint inhibits the entry stage of HCV lifecycle, likely by interacting with CD81.

To determine whether the lower level of HCV infection observed in EWI-2wint producing cells resulted from a reduced interaction between HCV glycoproteins and CD81, we compared the interaction between E1E2 and CD81 in Huh-7/pcDNA3.1 and Huh-7/EWI-2FLAG^fur^ cells, as detailed in Figure 1. Interestingly, our results demonstrated reduced interaction between E1E2 and CD81 in Huh-7/EWI-2FLAG^fur^ cells lysed in Bj/EDTA which conserves tetraspanins/partners interactions, as compared to cells lysed in TX/EDTA (Figure 6D). In contrast, the binding of E1E2 to CD81 was similar in Huh-7/pcDNA3.1 cells in both detergent conditions. These data indicate that the inhibition of HCV infection by EWI-2wint correlates with its capacity to inhibit the interaction between E2 glycoprotein and CD81.

**Figure 7:**
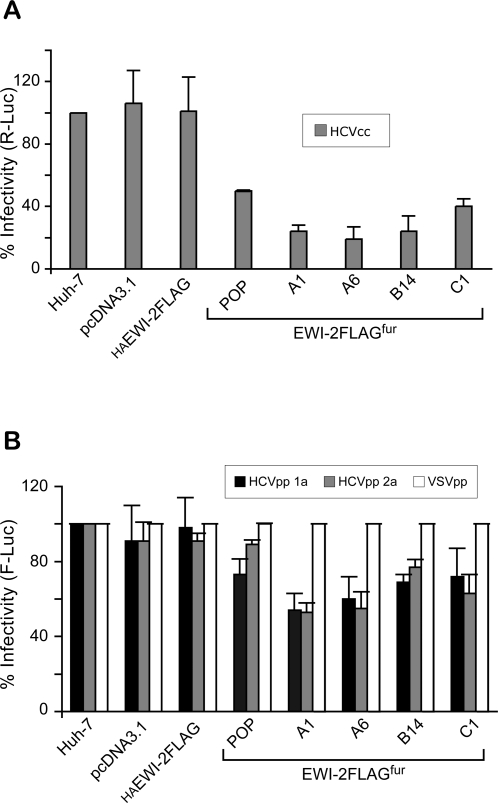
EWI-2wint inhibits the infection of both HCVcc and HCVpp. A, Cellular populations (pcDNA3.1, HAEWI-2FLAG and EWI-2FLAG^fur^ POP) and individual cellular clones (EWI-2FLAG^fur^ A1, A6, B14, C1) were infected with HCVcc expressing *Renilla* luciferase (R-Luc). In parallel, these cells were infected with HCVpp 1a, HCVpp 2a or VSVpp expressing firefly luciferase (F-Luc) (B), as described in Figure 6. At 2 days post-infection, cells were lysed and processed to measure the luciferase activity. The luciferase activities were normalized for protein concentration in each cell lysate. The results are presented as relative percentages to HCVcc (A) and HCVpp (B) infectivity on Huh-7 cells. HCVpp infections (B) were also normalized to VSVpp infections on Huh-7 cells. Results are reported as the mean±S.D. of three independent experiments.

In order to carry out statistical analyses of EWI-2wint effect on HCV infection, we next generated JFH1-based *Renilla* luciferase (R-Luc) reporter HCVcc and infected Huh-7 cells that lacked or expressed EWI-2wint (Figure 7A). Cellular populations (pcDNA3.1, HAEWI-2FLAG and EWI-2FLAG^fur^ POP) and individual cellular clones (EWI-2FLAG^fur^ A1, A6, B14, C1) have been used in these experiments. In parallel, we infected these cells with HCVpp 1a, HCVpp 2a or VSVpp (Figure 7B), as described in Figure 6. Our results showed that EWI-2wint expression inhibits HCVcc infectivity up to 80% (Figure 7A; EWI-2FLAG^fur^ A6) confirming our previous findings (Figure 5). It is noteworthy that EWI-2wint was less effective, reducing HCVpp infectivity only by 40–50% (Figure 7B; EWI-2FLAG^fur^ A1), which might be due to slight differences between HCVpp and HCVcc in the entry stages, as recently reported by others [31]. Once again, this study shows that EWI-2wint expression in Huh-7 cells leads to a significant reduction of HCV infection.

## Discussion

Here, we identified a novel CD81 associated protein, EWI-2wint, which is able to inhibit HCV entry into target cells by blocking the interactions between HCV glycoproteins and CD81. This finding indicates that the early steps of HCV entry into its target cells involve tight control of CD81 accessibility to the viral particle.

EWI-2wint may inhibit HCV entry by reducing E1E2-CD81 interactions for a number of possible reasons. EWI-2wint may reduce CD81 accessibility to envelope glycoproteins by steric hindrance. Alternatively, the association of EWI-2wint with CD81 may induce conformational modifications in CD81, blocking the binding of HCV heterodimers. Although the exact role of CD81 in the course of virus infection is not well defined, it is a key molecule required for productive infection by HCVpp and HCVcc of Huh-7 cells [3], [4], [5], [6], [7], [10], [32]. Moreover, most recently CD81 was shown to be required for the infection of primary hepatocytes by serum-derived HCV [33]. Previous studies have suggested that CD81 may not be required for the first attachment step, but rather function as a post-attachment entry co-factor [34], [35], [36]. Very recently, it has been shown that CLDN-1, 6 and 9 membrane proteins localized at tight junctions, are additional entry factors for HCV [11], [12], [37], indicating that HCV entry might involve a complex multistep process. CD81 might potentially be required for a post-binding step such as escorting the particle into the endocytic pathway or priming it for the pH triggered fusion mechanism. The association of EWI-2wint with CD81 could block such entry stages. Since our preliminary results suggest that CD81 and EWI-2wint likely colocalize in an endosomal compartment (unpublished data), EWI-2wint might exert its inhibitory effect on CD81 functionality in the endosomes. In addition, EWI-2wint may interfere with actin polymerization potentially required for HCV entry. Indeed, HCV replication requires microtubule and actin polymerization [38] and CD81 engagement leads to actin rearrangement [39], [40]. Furthermore, it has also recently been shown that CD81 and EWI-2 interact with Ezrin, an actin-linking ERM protein [23]. Lastly, we cannot exclude the possibility that EWI-2wint blocks other signaling pathways necessary for viral entry.

EWI-2wint is a cleavage product of the EWI-2 protein. The proteolytic cleavage of EWI-2 Ig1 domain occurs downstream of the RGR amino acids. A site directed mutagenesis analysis showed that RXR (where X is any amino acid) correspond to the consensus site recognized by the protease involved in this cleavage (unpublished data). Further analyses using protease inhibitors and small interference RNAs should enable us to identify this protease. Pulse-chase experiments in CHO cells showed that the cleavage occurs after N-glycan maturation (unpublished data), suggesting that it likely involves a Golgi resident protease. We showed that a RGRR furin cleavage motif made by insertion of an Arg residue into the EWI-2 sequence allows efficient production of EWI-2wint and additional cleavage product(s) in Huh-7 cells. As shown here, EWI-2 can be cleaved at RGR site to produce EWI-2wint, and it is likely that a furin-like protease may be responsible for this cleavage. Such a protease may be expressed differently among cell types, such that liver cells lacking the enzyme are consequently more sensitive to HCV infection. On the other hand, the accessibility of EWI-2 to protease(s) may be cell-type specific and might be modulated by other components of the tetraspanin webs that vary in different cell types [14], [16].

Although several molecules have already been implicated in the entry process of HCV, it is likely that additional specific factors regulate its entry into hepatic cells [41]. Silencing of endogenous EWI-2 in Huh-7 cells did not significantly affect HCVcc infection (data not shown) indicating that while being a major partner of CD81, EWI-2 is likely not involved in the HCV entry process. Here, we showed that EWI-2wint, present in some cell lines and absent from hepatic cells, is able to inhibit HCV entry. The production of a mAb directed against EWI-2wint will be essential to further analyse the expression profile of EWI-2wint in various tissues and cell lines. Nevertheless, our results suggest that, in addition to the presence of specific entry factors in the hepatocytes such as CD81, SR-BI, CLDN-1 and additional unidentified molecule(s), the absence of a specific inhibitor can also contribute to the hepatotropism of HCV. Interestingly, 293T cells, that are permissive for HCV entry when they express CLDN-1 [11], do not express EWI-2wint (Table 1). Further experiments with HCV non permissive cells expressing all known positive entry factors will be necessary to determine if the silencing of endogenous EWI-2wint is sufficient to induce HCV permissivity.

In conclusion, our observation constitutes a basis for the rational design of new antiviral molecules. Moreover, it represents a new mechanism that contributes to the regulation of the entry of a pathogen into a host cell.

## Materials and Methods

### Cells and antibodies

U937-CD81 cells have been described previously [42]. Primary hepatocytes were isolated from three human foetal livers (Advanced Bioscience Resources, Inc, Alameda, CA). HepG2-CD81 [10] have been described previously and were kindly provided by F-L. Cosset.

The mAbs used in this study included: 5A6 (anti-CD81 [43]), 8A12 (anti-EWI-2 [18]), 1F11 (anti-EWI-F [19]), H53 (anti-HCV E2 [44]), 3/11 (anti-HCV E2, kindly provided by J. McKeating [45]), ACAP27 and 486-D39 (anti-HCV core and HCV NS3, respectively, kindly provided by JF Delagneau [46]). The anti-beta actin (clone AC-15), the M2 anti-FLAG epitope mAb and the M2 anti-FLAG affinity gel were from Sigma. The HA11 (anti-HA, Clone 16B12) mAb was from Covance. The anti-claudin-1 (JAY.8) was from Zymed Laboratories. The anti-SR-BI (Cla-1) was from BD Transduction Laboratories.

### Plasmids

pcDNA3.1/CD81 plasmid has been described previously [18]. EWI-2 was FLAG-tagged (DYKDDDDK) at its C-terminus by polymerase chain reaction (PCR) amplification from template pCMVSport6/EWI-2 [18] and subcloned into a pcDNA3.1 vector (pcDNA3.1/EWI-2FLAG). Three Gly residues were introduced between EWI-2 and FLAG epitope sequences to increase epitope accessibility. Plasmid pcDNA3.1/wint-FLAG encoding EWI-2 signal peptide (amino acids 1–25) and EWI-2 amino acids 166–613 fused to FLAG tag was made by fusion-PCR from template pcDNA3.1/EWI-2FLAG. pcDNA3.1/HAEWI-2FLAG encodes EWI-2 signal peptide followed by HA9 epitope (YPYDVPDYA) tag fused to the entire sequence of EWI-2FLAG. Three Gly residues were also introduced between HA epitope and EWI-2FLAG sequence. In pcDNA3.1/EWI-2FLAG^fur^ plasmid, an Arg residue was introduced between EWI-2 amino acids 165 and 166. Cloning details and oligonucleotide sequences are available upon request.

### Cell transfection

CHO cells were transfected using ExGen500 (Eurogentec). CHO/CD81+EWI-2FLAG cell line was obtained by selection for 2 weeks with 500 µg/ml hygromycin and 800 µg/ml neomycin. The resulting cells were maintained in selection media as a polyclonal population. For stably transfected Huh-7 cells, cells were electroporated using the Gene Pulser apparatus (Bio-Rad) and neomycin was added 48 hours post-transfection at 600 µg/ml. After 2 weeks, isolated clones were split using selection cylinders and maintained in neomycin medium.

### CD81-E1E2 interaction assay

COS-7 cells were infected with a recombinant adenovirus (Ad) expressing the HCV proteins E1, E2, p7 and NS2 (Ad/E1E2p7NS2, amino acids 171–1026) [27] at a multiplicity of 25PFU per cell. At 48 h post-infection, cells were washed twice with ice-cold Dulbecco's phosphate-buffered saline (D-PBS), and lysed in one of the following lysis buffers : 1% Brij97 in D-PBS with calcium and magnesium (Bj/CaMg) or with 2 mM EDTA (Bj/EDTA), 1% Triton X-100 in D-PBS with calcium and magnesium (TX/CaMg), or with 2 mM EDTA (TX/EDTA). Clarified lysates were then incubated with mAb H53 pre-adsorbed onto rabbit anti-mouse protein A-sepharose overnight at 4°C (beads-E1E2). In parallel, rabbit anti-mouse-protein A beads were incubated with mAb 5A6 (beads-CD81) or an irrelevant control mouse mAb (beads-Cont). Cell lines were lysed in Bj/CaMg, Bj/EDTA, TX/CaMg or TX/EDTA lysis buffer at 10×10^6^ cells/ml, clarified and incubated with beads-E1E2, beads-CD81 and beads-Cont for 6 h at 4°C. After rinsing five times with the lysis buffer, complexes were eluted in non-reducing sample buffer, resolved by SDS-PAGE, and immunoblotted with mAb 5A6.

### Detection of cell surface biotinylated proteins

Cells were biotinylated as previously described [19] with 0.2 mg/ml EZ-link-Sulpho-NHS-LC-biotin (Pierce), and lysed into Bj/EDTA or TX/EDTA containing protease inhibitors (Complete, Roche). Lysates were precleared for 2 h at 4°C with protein A-sepharose (Amersham Biosciences) then incubated with mAbs pre-adsorbed onto rabbit anti-mouse-protein A beads for 2 h at 4°C. After rinsing, complexes were eluted, resolved by SDS-PAGE, and immunoblotted with peroxidase-conjugated streptavidin (Vector).

### EWI-2 silencing

To silence the expression of endogenous EWI-2 protein, Daudi cells were electroporated with negative siRNA directed against the FLAG epitope (5′ AUUACAAGGACGACGAUGA 3′, Dharmacon) and EWI-2 siRNA directed against the sequence 5′ GUUCUCCUAUGCUGUCUU 3′ (Dharmacon) corresponding to nucleotide sequence 253–270 of the ORF of EWI-2 [23]. Daudi cells were electroporated at 4°C using the Gene Pulser apparatus (400V, 950uF). Seventy two hours post-transfection, cells were biotinylated, lysed in Bj/EDTA and immunoprecipitated with 5A6 and 8A12 mAbs. Immunoprecipitated proteins were revealed by Western blotting with peroxidase-conjugated streptavidin or 5A6 mAb.

### Flow cytometry analysis

After trypsinization, transfected cells were fixed with Formalin Solution (formaldehyde 4%, Sigma) and permeabilized with PBS 0.2% BSA 0.05% Saponin. After rinsing with PBS 2% BSA 2 mM EDTA, cells were incubated 1 h at 4°C with 486-D39 anti-NS3 mAb. After rinsing with washing solution, cells were incubated with PE labeled goat anti-mouse (BD Pharmingen) for 45 min at 4°C, then washed and fixed with Formalin Solution. For CD81 staining, cells were detached with PBS 2 mM EDTA and incubated 1 h at 4°C with 5A6 anti-CD81 mAb. After rinsing with washing solution, cell lines were incubated with PE labeled goat anti-mouse for 45 min at 4°C, then washed and fixed with Formalin Solution (PFA 10%, Sigma). Cells stained only with the secondary antibodies were used as negative control. Labeled cells were analyzed using a FACS Beckman EPICS-XL MCL.

### Protein purification, mass spectrometry and N-terminal sequencing

For purification of EWI-2FLAG and wint-FLAG, ∼2×10^8^ CHO/CD81+EWI-2FLAG cells were lysed in 20 mM Tris-HCl, 150 mM NaCl, pH 7.6 (TBS), 1 mM EDTA and 1% Triton X-100 in the presence of protease inhibitors. Clarified cell lysates were precleared for 2 h at 4°C with protein A-sepharose and then incubated with an anti-FLAG M2 affinity gel for 2 h at 4°C. After five washes with TBS containing 0.2% Triton X-100 (TBS-T), FLAG-tagged proteins were eluted with 100 µg/ml FLAG peptide (Sigma) in TBS-T. Purified proteins were then precipitated with 5 volumes of acetone, incubated overnight at −20°C, and centrifugated. After rinsing with 80% acetone, proteins were resuspended in sample buffer, and resolved by SDS-PAGE. Colloïdal blue stained bands corresponding to EWI-2FLAG and wint-FLAG were then excised from the gel, reduced, alkylated with iodoacetamide (10 mg/ml in NH4HCO3, 20 mM) and digested overnight with 50 ng trypsin (Promega) in 20 mM NH4HCO3, or 100 ng V8 endoproteinase Glu C (Roche) in 0.1 M phosphate buffer pH7.7. The resulting peptide mixture was eluted from the gel, desalted, and spotted on a Maldi plate with freshly dissolved α-cyano-4-hydroxycinnaminic acid (5 mg/ml in 50% CH3CN, 20 mM citric acid). The dried spot was then washed with 20 mM di-ammonium citrate pH 4.5. Mass spectrometry was performed with a MALDI-TOF Voyager-DE-STR (Applied Biosystems). Spots were analysed by setting the following parameters: positive and reflector modes, acceleration voltage of 20KV, grid voltage of 61%, 90 ns of delayed extraction, low mass gate 500 amu. The laser energy required to desorb/ionise the samples was kept at low value, compatible with a good signal/noise ratio. Spectra were calibrated externally by using the [M+H+] monoisotopics ions from trypsinized lyzozyme. The theoretical list of digested peptides was obtained using MS-Digest http://128.40.158.151/ucsfhtml3.4/msdigest.htm. For N-terminal sequencing, purified proteins were transferred to PVDF and stained with amido black. Blotted EWI-2FLAG and wint-FLAG were sequenced in a Procise 492 sequanator (Applied Biosystems) using pulsed-liquid method.

### JFH-1 infection

The plasmid pJFH1, containing the full-length cDNA of JFH1 isolate (genotype 2a) and kindly provided by T. Wakita (National Institute of Infectious Diseases, Japan), was used to generate HCVcc as described [6], [47]. Infectious titers of viral stocks were estimated between 10^5^ and 10^6^ focus-forming units per ml, based on immunofluorescent detection of infected foci following infection of Huh-7 cells with serial dilutions of viral stocks. Cell lines were incubated with HCVcc (m.o.i. = 1) for 2 h at 37°C, washed and, incubated for additional 40 h at 37°C. Infections were scored by indirect immunofluorescence with anti-C mAb ACAP27 followed by Alexa^488^-conjugated goat anti-mouse (Jackson Immunoresearch). Fifteen micrograms of total protein were also analyzed by immunoblotting with 3/11 mAb followed by peroxidase-conjugated goat anti-rat immunoglobulins (Jackson Immunoresearch). The blots were then stripped and reprobed with an anti-beta actin followed by peroxidase-conjugated goat anti-mouse immunoglobulins (Sigma). Quantification of protein expression was performed using NIH Image 1.62 from the band mean densities.

To generate HCVcc expressing *Renilla* luciferase, we used the FL-J6/JFH-5′C19Rluc2AUbi genome [48] kindly provided by C.M. Rice. We replaced the region encoding the J6/JFH-1 HCV polyprotein with the CS-N6 JFH-1 sequence [47]. HCVcc were produced as described [6], [47], [48]. HCVcc were added to Huh-7 cells (m.o.i. = 1) seeded the day before in 24-well plates and incubated for 2 h at 37°C. The supernatants were then removed and the cells were incubated in DMEM 10% FBS at 37°C. At 40–48 h post-infection, *Renilla* luciferase assays were performed as indicated by the manufacturer (Promega).

### HCVcc replication analyses

*In vitro* transcribed full-length JFH1 RNA was transfected by electroporation as described [49]. At 40 h post-transfection, cells were analyzed by indirect immunofluorescence and flow cytometry.

### Indirect immunofluorescence microscopy

Infected or transfected cells grown on coverslips were fixed with 3% paraformaldehyde and permeabilized with 0.05% Triton X-100 in PBS. Cells were then stained with anti-C mAb ACAP27 followed by Alexa^488^-conjugated goat anti-mouse (Jackson Immunoresearch). Coverslips were mounted on glass slides using Mowiol, and observed with a Zeiss Axioplan 2 Axiophot 2 equipped with a 20×/1.3 numerical aperture lens. Fluorescent signals were collected with a Princeton cooled charged device using specific fluorescence excitation and emission filters. Images were processed with Adobe Photoshop software.

### Production of HCVpp and infection assays

HCVpp were produced as described previously [3], [50] with plasmids kindly provided by B. Bartosch and F.L. Cosset (INSERM U412, Lyon, France). For the production of VSVpp, a plasmid encoding the vesicular stomatitis virus glycoprotein G [51] was used. Supernatants containing the pseudotyped particles were harvested 48 h after transfection, filtered through 0.45-µm pore-sized membranes and conserved at 4°C. HCVpp were added to Huh-7 cells seeded the day before in 24-well plates and incubated for 2–3 h at 37°C. The supernatants were then removed and the cells were incubated in DMEM 10% FBS at 37°C. At 48 h post-infection, luciferase assays were performed as indicated by the manufacturer (Promega).
